# Recovery After Critical Illness: A Meta‐Ethnography of Patient, Family and Staff Perspectives

**DOI:** 10.1111/jan.70189

**Published:** 2025-10-02

**Authors:** Elizabeth King, Owen Gustafson, Annabel Williams, Francine Toye, Mark Williams, Sarah Vollam

**Affiliations:** ^1^ Faculty of Health, Science and Technology Oxford Brookes University Oxford UK; ^2^ Allied Health Professions Research & Innovation Unit Oxford University Hospitals NHS Foundation Trust Oxford UK; ^3^ Nuffield Department of Clinical Neurosciences University of Oxford Oxford UK; ^4^ Nuffield Department Orthopaedics, Rheumatology and Musculoskeletal Sciences University of Oxford UK; ^5^ Oxford University Hospitals NHS Foundation Trust Oxford UK; ^6^ Oxford Institute of Applied Health Research Oxford Brookes University Oxford UK; ^7^ NIHR Oxford Biomedical Research Centre Oxford UK

**Keywords:** critical care, family, patients, qualitative, rehabilitation, teamwork

## Abstract

**Aim:**

To synthesise primary qualitative studies reporting experiences of post‐hospital recovery for critical care survivors, their family and the healthcare professionals supporting them with a particular focus on physical impairment.

**Design:**

The review was conducted through a meta‐ethnography using the seven stages of Noblit and Hare.

**Methods:**

Qualitative studies or mixed‐method studies which included qualitative research were included if they were based on the phenomenon of interest. Study quality was assessed using the Critical Appraisal Skills Programme checklist and confidence in the findings with the GRADE CERQual framework.

**Data Sources:**

Five electronic databases (PubMed, EMBASE, CINAHL, AMED and PEDro) were searched from inception to February 2022 and updated in November 2024. Grey literature for primary qualitative studies was also searched.

**Results:**

A total of 26,249 studies were initially screened, and 38 eligible studies were analysed. Four themes were distilled describing the experiences of critical care survivors, their family members and staff involved in their care: ‘I survived, but I didn't thrive’, ‘Healthcare was there to save my life, but not for my long‐term recovery’, ‘I am a burden on my family, and they feel the weight of carrying me’ and ‘My body still doesn't work like it used to’.

**Conclusion:**

This meta‐ethnography is unique in bringing together the experiences of patients recovering from critical illness, their families, and the staff who support them after hospital discharge. Ongoing diverse physical impairments prevented patients from thriving, significantly impacting family members. All groups clearly identified unmet rehabilitation needs following critical illness.

## 1 Introduction

An increasing proportion of patients are surviving critical illness, and the focus of their survivorship should be on living full lives (Iwashyna and Speelmon 2016). More than half of critical care survivors experience at least one or more elements of post intensive care syndrome (PICS) (Mikkelsen et al. 2020). Post Intensive Care Syndrome (PICS) problems defined ‘as new onset or worsening of impairment(s) in physical, cognitive and/or mental health that arose after the ICU and persisted beyond hospital discharge’ (Inoue et al. 2019). The impact of these problems is profound as patients struggle to function in daily life (Inoue et al. 2024). These problems lead to increased mortality rates, reduced employment levels (with financial impact) and increased healthcare utilisation (Inoue et al. 2024; Ramnarain et al. 2021; Yanagi et al. 2021). There is also a clear impact on family members (Van Beusekom et al. 2016).

Recovery is the concept of ‘a *unique process of changing one's attitudes, values, feelings, goals skills, and/or roles to way of living a satisfying, hopeful and contributing life even with the limitations caused by illness’* (William 1993). Historically, applications of this concept were applied to fields of psychiatric and mental health care, yet they are fitting to encompass physical health as well (Roe et al. 2007). The meaning of recovery is different for each person with focus on gaining or recapturing meaning and purpose in life. Rehabilitation is regarded as a set of interventions to address the impact of health conditions by optimising function and reducing experience of disability. Rehabilitation interventions may facilitate recovery, although consideration should be given to rehabilitation potential as to whether patients are able to return to their normal or they gain a new baseline following critical illness (Nagi 1964).

Discharge from hospital into the community is an important transition. Post‐hospital rehabilitation following critical illness is multifaceted. To date, physical rehabilitation has focused on pulmonary rehabilitation style interventions or single interventions aimed at increasing strength, which have failed to demonstrate a sustained improvement in physical function (Connolly et al. 2016; McDowell et al. 2017; Shelly et al. 2017; McWilliams et al. 2016; Battle et al. 2019).

Primary qualitative research has focused on patient and family experiences of rehabilitation within the intensive care unit (ICU) and the transition of care into the community (Corner et al. 2019), capturing patients' vulnerability as they reconstruct their lives and reintegrate into their family (Vester et al. 2022). Although no qualitative studies have exclusively focused on the experience of physical impairment following critical illness, this is often explored within general studies of experiences of post‐ICU recovery. Family members describe the physical and emotional toll as they care for loved ones (Davidson and Harvey 2016). Understanding the perspectives of patients, family members and healthcare professionals has the potential to inform the delivery of effective rehabilitation after critical illness.

A qualitative evidence synthesis (QES) brings together the findings of primary qualitative studies. To date, five qualitative systematic reviews have explored the speciality of adult critical care. Kean et al. (2021) theorised the principle of survivorship in relation to critical illness, which highlighted the process of ‘moving on’ suggesting transitionary processes and working towards a sense of new normality. Hashem et al. (2016) categorised important themes of patient‐centred outcomes following critical illness. These themes encompassed satisfaction with life, mental health, physical health and ability to participate in social roles and activities domains. Rea et al. (2023) synthesised post‐hospital recovery at a patient and system level, recognising the uncertainties of recovery and the contribution of baseline health, suggesting the need for individualised care and recognition of co‐morbidities. Goddard et al. (2024) synthesised the experience of recovery and rehabilitation after hospital discharge and emphasised the lack of momentum, underpinned by vulnerability, physical weakness and boredom. Stewart et al. (2025) provided a framework of overarching and specific principles regarding ICU follow‐up clinic and rehabilitation. Whilst physical function was acknowledged, an emphasis was on multicomponent bundles of care such as practicalities, co‐ordination and communication with primary care. One review focused on survivorship and identified the key stakeholders within it, such as the patient, family and healthcare systems. In summary, these qualitative syntheses focus on survivorship and the post critical care period as a journey of recovery with varying challenges, support needs and constructs aligning to the biopsychosocial sphere.

## 2 The Review

### 2.1 Aim(s)

We aimed to synthesise primary qualitative studies reporting experiences of post‐hospital recovery for critical care survivors, their families and the healthcare professionals supporting them with a particular focus on physical impairments.

## 3 Methods/Methodology

### 3.1 Design

We undertook the seven stages of meta‐ethnography for synthesising qualitative research developed by Noblit and Hare (1988). We followed the eMERGe Reporting Guidance for reporting meta‐ethnography (France et al. 2019), published the study protocol (King et al. 2022), registered the review on PROSPERO and presented the search results in a PRISMA flow diagram (Page et al. 2021).

A meta‐ethnographical approach was chosen for this study to provide conceptualisation of the phenomenon, the inductive and interpretative nature and the development of a conceptual model. The use of a meta‐ethnographical approach is utilised in healthcare practice and policy as new evidence is generated from the synthesis of data from multiple studies (Atkins et al. 2008).

#### 3.1.1 Stage One—‘Getting Started’

The aims and rationale were developed by EK, MW, AW, SV and OG. Preliminary searches were undertaken, including in PROSPERO, and no current or underway qualitative systematic reviews on the topic were identified.

### 3.2 Search Methods

#### 3.2.1 Stage Two—‘Deciding What Is Relevant’

We searched five databases: PubMed, EMBASE, CINAHL, AMED, PEDro from inception to February 2022 and updated the searches in November 2024. We also searched grey literature through trial registries, pre‐print servers and Google Scholar.

We used Medical Subject Headings with free text terms, including variations of ‘intensive care’ and ‘qualitative’. The search term of ICU was utilised to assess relevant papers despite the focus being on post‐ICU post‐hospital care, which remains in its infancy as a direct field of literature. We used the STARLITE mnemonic (sampling strategy, type of study, approaches, range of years, limits, inclusion and exclusions, terms used and electronic sources) (Booth 2006) to report our search strategy (Table 1). Given the lack of studies focusing specifically on physical impairment, we did not include this in our search terms, aiming to identify studies which may have considered the impact of this within broader studies.

**TABLE 1:** STARlite.

S: Sampling	Comprehensive
T: Types of studies	Qualitative or mixed methods with qualitative, fully reported
A: Approach	Electronic databases with hand searching
R: Range of years	No limits
L: Limits	English language
I: Inclusion and exclusion	Inclusion Adult critical illness survivors Family members who supported or care for adult critical illness survivors Healthcare professionals signposting to or providing rehabilitation for adult critical illness survivors (nil limit to staff groups) Exclusion Adolescents (under 18 years of age) Paid caregivers to patients of this interest
T: Terms used	Pubmed–Critical care OR Critical illness OR Intensive care OR ICU or ICUs or ITU OR Adult respiratory distress syndrome OR ARDS OR Critical* ill* AND Qualitative OR Phenomenlog* OR Hermeneutic* OR Husserl OR Heidegger OR Ethnog*
E: Electronic databases	PubMed, EMBASE, CINAHL, AMED, PEDro

### 3.3 Inclusion and/or Exclusion Criteria

#### 3.3.1 Stage Two—‘Deciding What Is Relevant’

We included qualitative studies or mixed‐method studies which included qualitative research published in English. Qualitative findings were only extracted from mixed‐method studies.

We included studies that explored the experiences of adult critical illness survivors beyond hospital discharge, their family members and healthcare professionals (with no limit to staff groups). We kept the inclusion criteria broad to reflect the heterogeneity of ICU populations.

We excluded studies exploring the experiences of adolescents (under 18 years of age) or professional caregivers.

### 3.4 Search Outcome

Duplicate studies were removed within Rayyan software (Ouzzani et al. 2016). Two independent reviewers (EK and OG) then screened search results at title, abstract and full text for potential studies. Any studies that did not fulfil the inclusion criteria were disregarded, and MW resolved any disagreements between EK and OG.

### 3.5 Quality Appraisal

We used the ten Critical Appraisal Skills Programme (CASP) questions for appraising qualitative research to assess study quality (Long et al. 2020). Whilst there is no consensus on quality appraisal frameworks, CASP is endorsed by Cochrane and the World Health Organisation for use in meta‐ethnography (Cargo et al. 2018; Hannes and Macaitis 2012). Two reviewers (EK and OG) independently appraised each included study, and disagreements were negotiated between them until consensus was reached.

We assessed our confidence in each review finding using the GRADE‐CERQual framework. This consists of four domains: (1) ‘methodological limitations’ of primary studies, (2) ‘relevance’—the extent to which the primary studies are applicable to the review aims, (3) ‘adequacy of data’—the quantity and richness of data supporting each finding and (4) ‘coherence’—how clear and coherent the data between the primary studies and the review findings. An overall assessment was made by EK, MW, AW and SV based on the four domains to assess the confidence of each finding to be high, moderate, low or very low (Lewin et al. 2018).

### 3.6 Data Abstraction

#### 3.6.1 Stage Three—‘Reading Included Studies’

One reviewer (EK) extracted study characteristics information (such as country of origin and participant groups) to facilitate discussion of comparison and transferability of findings. We also extracted the time point of data collection. Data was extracted into an Excel database.

### 3.7 Synthesis

#### 3.7.1 Stage Four—‘Determining How Studies Relate’

Three reviewers (EK, SV and AW) read three studies and extracted the primary research findings before distilling the meaning of each finding into simple active voice English. Following discussion and agreement regarding clarity of the distillation, one reviewer (EK) completed this for the remaining studies.

#### 3.7.2 Stages Five and Six—‘Translating Studies Into Each Other’ and ‘Synthesising Translations’

One reviewer (EK) coded the extracted data by assigning a descriptive label and these were iteratively developed into themes with the research team (MW, SV and AW). Data from patients, family and healthcare professionals were analysed separately to explore differences between groups. We then looked for similarities and differences between these groups to develop our final themes, through a process of constant comparison. We discussed and refined these themes with a fourth researcher (FT) who had experience of meta‐ethnography analysis. We used NVivo 12 to assist in coding and organisation of data.

#### 3.7.3 Stage Seven—Expressing the Synthesis

When all reviewers agreed on the final themes, we developed a conceptual model through discussion between all authors and related our model to existing literature.

### 3.8 Reflexivity

The research team had a range of professional and research experiences. EK is a physiotherapist who works within an ICU follow‐up clinic. OG is a physiotherapist with experience treating patients within and post ICU and is researching the long‐term consequences of critical illness, specifically the musculoskeletal health state. SV is a nurse researcher with a particular interest in detecting and managing clinical deteriorations in hospitalised patients and post‐ICU ward management. AW is a physiotherapy lecturer with an interest in qualitative research and has previously worked in intensive care. MW is a physiotherapist with a musculoskeletal background and a researcher with an interest in rehabilitation interventions. FT is a qualitative researcher and anthropologist and was sought for expert advice on qualitative methodology and analysis. We aimed to challenge and develop analytic decisions rather than aim for consensus during the stages of this meta‐ethnography.

### 3.9 Findings

**FIGURE 1:**
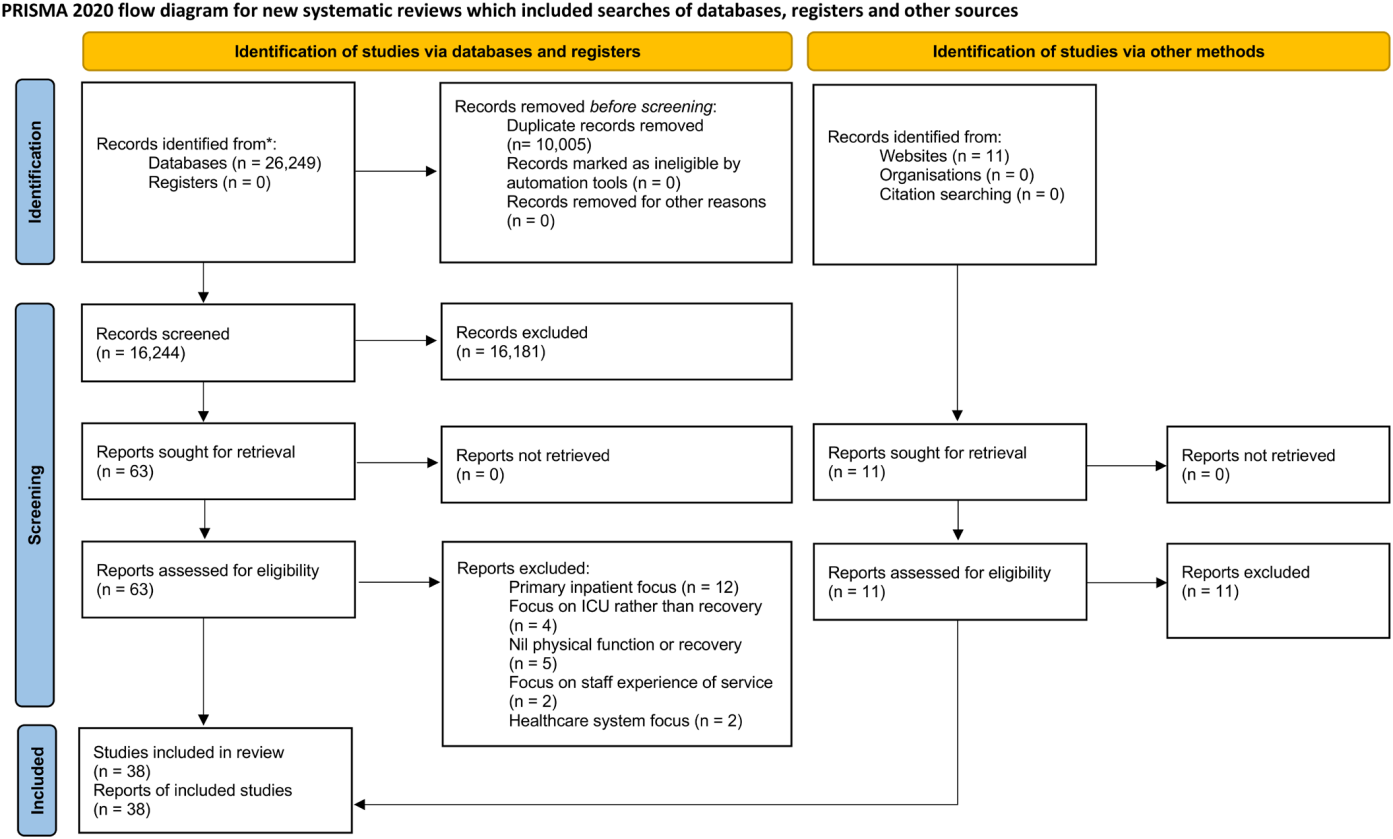
PRISMA flowchart of the meta‐ethnography.

We identified and screened 26,249 potentially relevant papers and 38 qualitative studies that satisfied all inclusion criteria (Vester et al. 2022; Cox et al. 2009; Pattison and Dolan 2009; Prinjha et al. 2009; Karlsson et al. 2016; Frivold et al. 2016; Palesjö et al. 2015; Pattison et al. 2015; Ågård et al. 2015, 2012; Czerwonka et al. 2015; Abdalrahim and Zeilani 2014; Deacon 2012; Nelderup and Samuelson 2020; Page et al. 2019; Kang and Jeong 2018; Nelderup et al. 2018; Rohr et al. 2021; Allum et al. 2018; Alexandersen et al. 2021; Major et al. 2021; Walker et al. 2015; Hanifa et al. 2018; Jensen et al. 2017; Geense et al. 2021; Calkins et al. 2021; Gehrke‐Beck et al. 2021; Thurston et al. 2020; Sevin et al. 2021; Johansson et al. 2004; Eaton, Lewis, et al. 2023; Eaton, Danesh, et al. 2023; Juuso et al. 2024; Danielis et al. 2024; Zhang et al. 2024; Wendlandt et al. 2024; O'Neill et al. 2024; Paton et al. 2024). For full details, see Figure 1: PRISMA flow diagram. Twenty studies explored patient experiences, six explored family member experiences, 11 included dyads of patient and family members, and five explored staff experiences. The selected studies incorporated 479 patients, 196 family members and 122 staff across a variety of international geographical locations, predominantly the USA, Europe and UK (see Table 3). Data collection time points ranged from 1 month to 11 years after hospital discharge. Study quality was generally good, with only one domain (adequate consideration of the researcher/participant relationship) commonly not met (Table 2). Confidence in one theme was rated as high, and three themes were rated as moderate confidence (see Data S1).

**TABLE 2:** Study appraisal using the CASP tool.

	Clear statement of the aims?	Is qualitative methodology appropriate?	Was the research design appropriate to address the aims?	Was the recruitment strategy appropriate?	Was the data collected in a way that addressed the issue?	Researcher/participant relationship adequately considered?	Have ethical issues been taken into consideration?	Was the data analysis sufficiently rigorous?	Is there a clear statement of findings?	How valuable is the research?
Cox et al. (2009)										
Pattison and Dolan (2009)										
Prinjha et al. (2009)										
Karlsson et al. (2016)										
Frivold et al. (2016)										
Palesjö et al. (2015)										
Pattison et al. (2015)										
Ågård et al. (2015)										
Czerwonka et al. (2015)										
Abdalrahim and Zeilani (2014)										
Ågård et al. (2012)										
Deacon (2012)										
Nelderup and Samuelson (2020)										
Page et al. (2019)										
Kang and Jeong (2018)										
Nelderup et al. (2018)										
Rohr et al. (2021)										
Allum et al. (2018)										
Alexandersen et al. (2021)										
Major et al. (2021)										
Walker et al. (2015)										
Hanifa et al. (2018)										
Jensen et al. (2017)										
Geense et al. (2021)										
Vester et al. (2022)										
Calkins et al. (2021)										
Gehrke‐Beck et al. (2021)										
Thurston et al. (2020)										
Sevin et al. (2021)										
Johansson et al. (2004)										
Eaton, Lewis, et al. (2023) _examining										
Eaton, Danesh, et al. (2023) _importance										
Juuso et al. (2024)										
Danielis et al. (2024)										
Zhang et al. (2024)										
Wendlandt et al. (2024)										
O'Neill et al. (2024)										
Paton et al. (2024)										

*Note:* Key: Green, yes; orange, cannot tell; red, no.

**TABLE 3:** Characteristics of studies.

Author, year	Country	Sample (*n*)	Data collection	Data collection time points	Aim of study
Cox et al. (2009)	USA	Survivors (21) Mixed caregivers (24–5 spouse/partner, 2 child, 6 other family member, 1 friend)	Semi‐structured interviews	Randomised to time points 1–3 months, 4–6 months, 7–9 months and 10–12 months after discharge	To understand the lived experience of critical illness in ARDS survivors.
Pattison and Dolan (2009)	UK	Patients (28)	Mixed method: interview and questionnaire	Up to 12 months post critical care discharge	To explore the impact of critical care experiences on patients' long‐term health
Prinjha et al. (2009)	UK	Patients (34)	Semi‐structured interviews	Up to 11 years	To explore patients' perceptions of ICU follow‐up services
Karlsson et al. (2016)	Sweden	Patients (15)	Interview: predetermined guide	2 months after ICU discharge	To explore and describe older patients' experiences of recovery and need of care within 2 months following discharge from hospital after being cared for in an intensive care unit
Frivold et al. (2016)	Norway	Patients closest relatives' (survivors and non) (13)	Interviews: open ended	3 months to 1 year after the discharge or death of an intensive care unit patient	To illuminate relatives' experiences of everyday life after a loved one's stay in an intensive care unit
Palesjö et al. (2015)	Sweden	Patients (7)	Interview: open ended	> 2 years at home post hospital discharge	To describe and interpret the essential meaning of lived experiences of being in a critical illness‐recovery process after a life‐threatening condition as narrated more than 2 years after the critical event
Pattison et al. (2015)	UK	Patients (22)	Email interviews	1 month and 6 months after discharge from ICU	To explore the experience, needs and outcomes over time, of patients discharged from critical care
Ågård et al. (2015)	Denmark	Patient (18) Spouse (18)	Semi‐structured interviews	3 months and 12 months after discharge from ICU	To explore the challenges facing spouses of ICU survivors and describe and explain their concerns and caregiving strategies during the first 12 months post‐ICU discharge
Czerwonka et al. (2015)	Canada	ICU survivors (5) Family caregivers (7)	Semi‐structured interviews	Approximately 7 days (only 1 survivor), 3, 6, 12 and 24 months post‐ICU discharge	To delineate the needs and experiences of survivors of complex critical illness and their family caregivers across the illness and recovery trajectory using the TIR framework as a guide.
	Jordan	Patients (18)	Interview: open ended	3 months post discharge from ICU	To describe the experiences of Jordanian survivors of critical illnesses 3 months after discharge from a hospital ICU
Ågård et al. (2012)	Denmark	Couples (18)	Semi‐structured interviews. Focus groups	3 and 12 months post ICU discharge	To explore the challenges facing ICU survivors with a cohabiting spouse or partner and explain patients' concerns and coping modalities during the first 12 months post ICU discharge.
Deacon (2012)	England	Patients (35)	Online questionnaire. Open ended questions.	Not detailed	To explore former ICU patients' views on what the key components of a post ICU rehabilitation programme should be.
Nelderup et al. (2018)	Sweden	Partners (6)	Semi structured interviews	6–10 months after their partner's ICU admission	To explore the experiences of partners of intensive care survivors and their need for support after intensive care
Page et al. (2019)	England	Survivors (16) Family members (15)	In‐depth interviews	5–10 months post ICU discharge (with some family ones on ICU)	To understand the critical illness trajectory from patient and relative perspectives
Kang and Jeong (2018)	South Korea	Patients (13)	In‐depth interviews—semi structured	1–10 months post hospital discharge	Explored critical care survivors' experience of post‐intensive care syndrome
Nelderup et al. (2018)	Sweden	Patients (12)	Explorative interview study. Unstructured interviews with single open ended questions	2–6 months post ICU discharge	To explore intensive care survivors' experiences of recovery after hospital discharge, from the patient perspective
Rohr et al. (2021)	Germany	Staff (47) (mixed professional backgrounds, disciplines and healthcare sectors)	Semi‐structured focus groups and expert interviews	N/A	To capture the perspectives of health care providers on the development of the first intensive care unit follow‐up‐clinic in Germany.
Allum et al. (2018)	England	Patients (12)	Semi‐structured interviews	Not detailed	To describe patients' views on the types of support they feel are important in aiding recovery following critical illness.
Alexandersen et al. (2021)	Norway	Patients (17)	Semi‐structured interviews	6–20 months post ICU discharge	To provide knowledge about what promotes and challenges the salutogenic resources of inner strength and willpower in long‐term ICU patients back home after hospital discharge
Major et al. (2021)	Netherlands	Physios (11)	Focus groups	Not detailed	The aim of this study was to investigate the feasibility of an interdisciplinary home‐based intervention for patients with new or worsened impairments within one of the domains of PICS, initiated immediately after hospital discharge and targeting (physical) recovery and self‐management in comparison to patients receiving usual care
Walker et al. (2015)	England	Patients (16)	Focus groups	Not detailed	Gain a more in‐depth insight into patients' perceptions of their quality of life after hospital discharge and their experiences of aftercare services, be it usual care or the PIX exercise intervention programme
Hanifa et al. (2018)	Denmark	Patients (10)	(1) an observational study of the current follow‐up consultation; (2) a semi‐structured interview	Nil detailed	To describe former ICU patients' consultation experiences, specifically regarding content and setting. To explore the benefits of the consultation in regard to individual patients' symptoms of PICS
Jensen et al. (2017)	Denmark	Patients (12) Relatives (9) (partner, partner or child)	(1) audio‐recordings of consultations, (2) patient photographs and (3) completed reflection sheets	1–3, 4–5, 9–11 months	To describe the patient experience of ICU recovery from a longitudinal perspective by analysing follow‐up consultations at three time‐points.
Geense et al. (2021)	Netherlands		Semi‐structured interviews	1 year post ICU	To identify and purposively select ICU survivors, who experience a reduced QoL one year after ICU admission, for a follow‐up in‐depth qualitative study to get more insight into their daily functioning and their story behind the numbers
Vester et al. (2022)	Denmark	Patients (12) Relatives 7 (hereof six patients and six relatives were of the same family)	Semi‐structured interviews	Not detailed but post hospital d/c as sampled through the follow‐up clinic	To explore patients' and relatives' experiences of everyday life after critical illness
Calkins et al. (2021)	USA	Patients (18)	Semi‐structured interviews	At least 4 weeks post hospital discharge	To understand barriers and facilitators of recovery for critical illness survivors', who are discharged home from the hospital and do not have access to dedicated outpatient care.
Gehrke‐Beck et al. (2021)	Germany	Staff (14)	Semi‐structured interviews	N/A	To illuminate and understand the functioning of the intervention in the social background of a GP practice and to extract suggestions for future and optimised aftercare in general practice
Thurston et al. (2020)	Australia	Patients (35)	Semi‐structured interviews	6.2 months after ICU discharge (range 5 to 7.4 months)	By exploring their perspectives and experiences we hoped to identify areas of importance to their recovery and how these may have shaped their self‐perceived outcomes
Sevin et al. (2021)	US, UK, Australia	Caregivers (20): 16 (80%) from the US, 2 (10%) from Australia, and 2 (10%) from the UK	Semi‐structured interviews	‘Various timepoints in recovery’	To elucidate caregiver needs in the critical illness recovery period, as well as components of post‐ICU programs that caregivers found beneficial
Johansson et al. (2004)	Sweden	Next of kins (14)	Interviews	3–15 months after the patients' discharge to the home	To generate a theoretical model with regard to relatives' coping when faced with the situation of having an adult NOK recovering at home after critical illness
Eaton, Lewis, et al. (2023)	USA	Patients (17)	Semi‐structured interviews	13–33 months following index ICU stay	To examine the needs of survivors of critical illness using a palliative care lens.
Eaton, Danesh, et al. (2023)	USA based but 15 international sites (Canada, United States, United Kingdom)	Clinicians (29)	Semi‐structured interviews	N/A	To explore how ICU recovery programs may influence clinician well‐being.
Juuso et al. (2024)	Sweden	Patients (13)	Interviews	3–6 months after hospital discharge	To elucidate meanings of recovery for people once critically ill with COVID‐19
Danielis et al. (2024)	Italy	Relatives (24)	Interviews	1–6 months post hospital discharge	To investigate the experiences of family members in the caregiving role following the discharge of their loved ones from an ICU, with a particular focus on how these experiences impact their quality of life
Zhang et al. (2024)	China	Clinicians (21)	Semi‐structured interviews	N/A	To explore and describe the barriers and facilitators post‐intensive care follow‐up services from the perspective of critical care professionals
Wendlandt et al. (2024)	USA	Caregivers (21)	Semi‐structured interviews	Greater than 6 months post ICU admission	To gain new, person‐centred insights into wellness and distress for caregivers of people with recent acute cardiorespiratory failure to reconceptualize the relevant post‐ICU caregiver outcomes and inform the development of effective support interventions for these caregivers
O'Neill et al. (2024)	Northern Ireland	Patients (15)	Semi‐structured interviews	6 and 12 months from ICU discharge	To explore the views of patients after discharge from ICU about their recovery at six and twelve months and factors that facilitated recovery, and to determine additional services that patients felt were missing during their recovery.
Paton et al. (2024)	Australia	Patients (20)	Semi‐structured interviews	6 months from ICU admission	To identify key concerns for ICU survivors during the recovery from critical illness through qualitative analysis.

The findings from the 38 studies were distilled into four themes: ‘I survived, but I didn't thrive’ ‘Healthcare was there to save my life, but not for my long‐term recovery’, ‘I am a burden on my family, and they feel the weight of carrying me’ and *‘*My body still doesn't work like it used to*’*. Each theme is described below, using narrative exemplars from the primary studies.

#### 3.9.1 I Survived, but I Didn't Thrive

This theme describes patients' uncertainty about their recovery and how they struggled to come to terms with their diminished levels of function and mobility. It incorporates the perceptions of family members who supported the transition from hospital and their reflections and expectations about how patients should restore their lives and wellbeing.

Being discharged from the acute hospital and the prospect of the recovery ahead was a critical time point for patients to reflect on their mortality. Some patients resigned themselves to a ‘new life’ in the context of illness or disability. This could lead to feelings of bitterness as some felt this was a punishment they did not deserve.

I am bitter, why would I be punished…I didn't ask to become ill. (patient, Palesjö et al. 2015)

Whilst feeling enormously grateful to have escaped ‘death's door’, patients and family members expected and wanted more than just survival. However, there was an underlying sense that healthcare professionals felt that patients should be grateful to be alive.

When we asked about the quality‐of‐life issues, I actually heard one of the doctors say, “Well, he's alive, isn't he?” (patient, Cox et al. 2009)

Despite having faced death and survived, some experienced profound losses to self. For example, some had lost trust and confidence in their bodies to support and protect them, meaning they were unable to perform everyday tasks. Others had to prioritise tasks of daily living, therefore limiting their involvement in more meaningful activities. Family members observed how the lack of physical strength limited their loved one's ability to engage in activities beyond very basic functions. Watching their loved one's struggle made family members sad and depressed.

…he sat there unable to do anything. He was so weak… it was awful. (spouse, Ågård et al. 2015)

The body didn't obey me, I was weak in my arms and legs and I couldn't climb the stairs. (patient, Palesjö et al. 2015)

Patients felt scared to leave the house and missed out on social engagement, leading to disconnection and isolation from the outside world. Some felt like they had lost their sense of purpose, exacerbating loss to identity.

I pretty much like staying indoors, staying out of shopping centers…I'm still scared to death I'm going to die. (patient, Thurston et al. 2020)

Family members felt that, given this new chance, patients should take the lead on restoring their lives and wellbeing given this new chance, although at the same time, recognising limitations due to ongoing ill health. Some recognised that loved ones might understandably lack motivation to take control, or that they might be just too overwhelmed by present experiences.

…if you are the survivor, you've got to take ownership of your wellbeing… and feel well enough that he wants to do it…he just doesn't feel well… (caregiver, Czerwonka et al. 2015)

#### 3.9.2 Healthcare Was There to Save My Life, but Not for My Long‐Term Recovery

This theme highlights how patients struggled to access follow‐up care and rehabilitation once they were discharged from hospital, and how families had to advocate for them. Healthcare professionals also described a lack of consensus on operational delivery and communication after hospital discharge.

Once patients had returned home, they and their families found it difficult to get information regarding their illness and treatments that they received whilst they were in hospital. Some struggled to access healthcare to support their recovery despite relentless attempts to make contact; this could absorb precious time whilst tired and often overwhelmed. Some were unsure about what to expect in terms of their recovery and did not know what to consider “normal” This left some feeling uncertain and uncared for. Family members struggled to advocate for their loved ones as they did not always know who to contact to seek support. Even when patients contacted their General Practitioner, they could not always confirm details of hospital admission and had to contact the hospital team.

It was frustrating that they had all these appointments made for me that I knew nothing about… I had one appointment that I cancelled because when I called I didn't know who it was with, what it was about and they said, ‘well you're scheduled for surgery because you have gallstones’, I said, I don't remember him telling me I had gallstones, so I cancelled it. (patient, Calkins et al. 2021)

[My GP] had to ring the hospital to find out exactly what happened and what was going on with me… (patient, Thurston et al. 2020)

Patients who were invited to attend an ICU follow‐up clinic were reassured and took comfort in understanding what had happened and how to adjust to their current life. Patients found it valuable to see staff who had cared for them whilst in ICU as they could personally relate to what they had been through and see how they had improved since.

I valued seeing the same healthcare professionals who remembered me and could see my progress as I recovered. (patient, Prinjha et al. 2009)

In one study, there was a sense that due to the high cost of extensive training for ICU clinicians, their time and input should remain within the walls of the ICU, rather than devoting time to patient care after discharge from hospital. This re‐emphasises the focus on saving lives, rather than ensuring restoring quality of life.

The cost of cultivating an ICU doctor or nurse is high…They should not divert their energy to follow‐up. (staff, Zhang et al. 2024)

There was no underlying sense of agreement amongst staff about which patients should be invited to clinic (beyond those in need of ongoing care) and at which time point. Some staff expressed concerns that clinics only provided screening for patients rather than treatment interventions. Unnecessary onward referral also created a delay in patient care and might inhibit continuity of care.

“I think such an ICU follow‐up clinic only makes sense if it can offer the actual treatment.” (staff, Rohr et al. 2021)

Some patients felt desperate about making very limited recovery and felt that they had to do something themselves to move forward. For example, some made the decision to pay privately for physiotherapy. Covering treatment costs could create a financial burden when covering costs for treatment, and only some families could fund private care. In countries without free healthcare provision, some worried that their insurance would not cover their needs.

I knew that whatever I had in my savings, it wasn't going to cover it… (patient, Eaton, Lewis, et al. 2023; Eaton, Danesh, et al. 2023)

#### 3.9.3 I Am a Burden on My Family, and They Feel the Weight of Carrying Me

This theme captures the deeply personal impact of patients describing themselves as a burden to their family. It also captures how family members physically and emotionally cared for their loved one and carried the emotional toll, while adjusting to new roles.

Some patients felt that they were a burden on family members, dependent on them for fundamental needs such as feeding and personal care, whilst one patient felt she was an inconvenience for her daughter.

Could you imagine what it's like to depend on someone else just to get through the day? To watch my wife get up earlier…take care of my bandages, my feeding tube… (patient, Cox et al. 2009)

… it's a hassle for my daughter because she has two children… She had to change her house around because of me. (patient, Eaton, Lewis, et al. 2023; Eaton, Danesh, et al. 2023)

Family members were struck with worry, anxiety and/or depression as they cared for their loved ones. Despite one family member being a nurse, she was overcome with concern of how they would manage if their loved one's condition deteriorated.

I am a nurse and see this every day… It is just too much sometimes, overwhelming. What will I do if he gets worse? We are sinking. (family, Cox et al. 2009)

Some considered the responsibility to care was theirs alone, to endure irrespective of significant changes in personal relationships. An example of this was having to tolerate shouting at by a family member who became dependent following hospitalisation, equating this to caring for an infant.

…he came after me, yelling…. It was just like having a newborn baby in the house, like having become the mother of an old man… (family, Johansson et al. 2004)

Whilst caring, family members gained a range of new skills often through trial and error. Others received intensive education from healthcare staff to ensure the provision of tangible, practical support. Some felt a deep sense of responsibility to ensure the needs of loved ones were met, and this level of responsibility could be burdensome.

Mom has a stoma, so basically, I have to change her bag every day because she can't do it for herself […] The nurse taught me there how to deal with the stoma … They called me one day to give me, like, an accelerated course all in one day. (family, Danielis et al. 2024)

Suddenly I got a lot of responsibilities, things I had never tried and I just didn't know what to do. It's easy to make mistakes and nobody else was there. (family, Ågård et al. 2015)

Beyond the emotional and practical impact of the caring burden, some family members experienced a physical cost. For example, a wife describes her back spasm whilst caring for her husband. Whilst spouses took over the care of the family and household chores, this meant that some patients began to question their personal role and identity.

I got a muscle spasm in my back, couldn't walk…he got very angry with me…. (family, Johansson et al. 2004)

I used to do everything…My husband still takes care of everything, leaving me wondering: What am I in this family? (patient, Vester et al. 2022)

Despite experiencing the devastation of their loved one being so unwell, family members suggested that sometimes their own need could be forgotten about in context of the overarching illness.

Because we always think only about the sick person, but in reality, it's the illness of a person that impacts their whole family. (family, Danielis et al. 2024)

#### 3.9.4 My Body Still Doesn't Work Like It Used to

This theme describes physical impairments that persisted following critical illness and the adjustments made to living with the long‐term consequences.

As patients endured problems such as long‐term pain and disability, some felt they were stuck at a very basic level of function with limited hope of return to work. This could mean financial problems.

I am almost at a subsistence level… I will never be able to return to work…when I'm not able to lift things… I do not sleep at night due to the pain… There are these two things, pain and economy. (patient, Alexandersen et al. 2021)

Some patients continued to struggle with loss of muscle strength and low energy even when they carefully followed recommendations for their recovery (e.g., an exercise regime). This could be very frustrating.

Even after being home for a year, and doing all my exercises, I was still not as strong and active as when I went into hospital. (patient, Ågård et al. 2012)

Patients who did not fully recover described how the legacy of being so unwell might now define them. Some had lost hope in ever recovering. At the same time, patients had to balance expectations of those around them and could feel pressure from others to make more progress, sometimes hiding negative feelings.

My medical condition is my life now. (patient, Cox et al. 2009).

You can't see it so it's easy to pretend it does not exist…. The high expectations come not only from family and friends but doctors as well. (patient, Deacon 2012)

### 3.10 Conceptual Model

**FIGURE 2:**
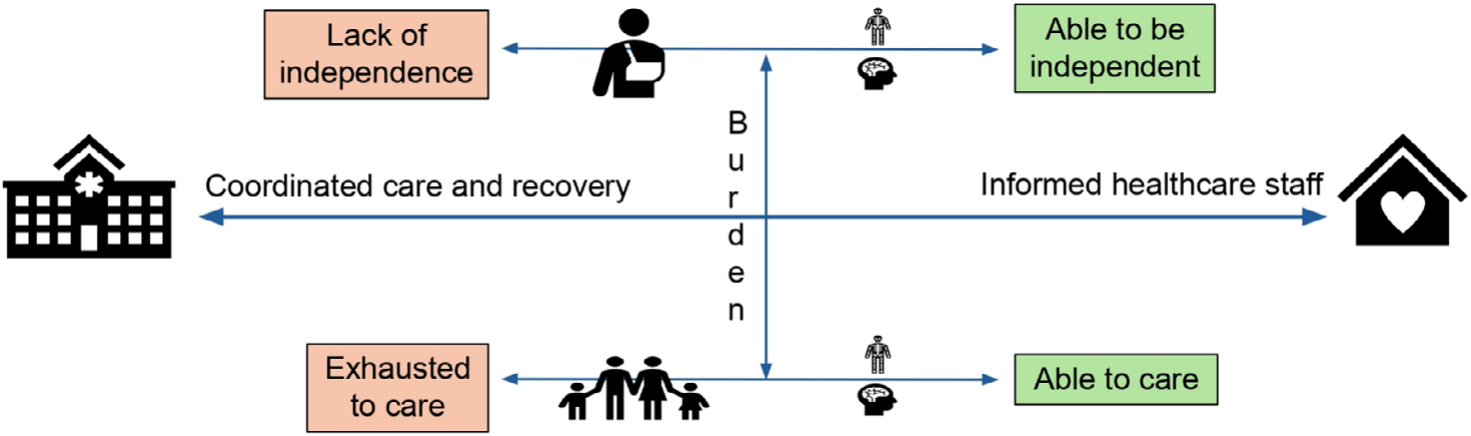
Recovery from hospital to independence.

Figure 2 illustrates our conceptual model, drawn from the data, illustrating the essence of the four themes and situating them on a trajectory of recovery from hospital to independence. Three horizontal domains represent the experiences of patients, healthcare staff and families. Co‐ordinated provision of care and rehabilitation is central to the path of recovery, across the continuum of patient recovery and family ability to care. The horizontal bidirectional arrows across the three domains of experience highlight the balance of progression and the risk of regression, given the complexities of the body and mind, and the burden of dependency.

This conceptual model adds to the narrative findings through a visual communication of concepts. It facilitates understanding, comparison and integration of the multi‐dimensional perspectives. By presenting the concepts from this synthesis through an illustrative model, it extends access to audiences such as clinicians who may be less familiar with reading literature.

## 4 Discussion

Our meta‐ethnography synthesises the experiences of long‐term recovery after critical illness from multi‐dimensional perspectives. Overall, experiences of critical care survivors highlight that they do not always thrive, and that they can become dependent on their family for basic needs. At times, they perceive themselves as a burden on their families, with families bearing the cost of providing physical and emotional care. Our findings show that the impact of physical impairments following critical illness was problematic and persistent, and rehabilitation was inhibited by a lack of dedicated follow‐up. These physical impairments are described as impacting complex functional tasks as well as manifesting as discrete problems such as muscle weakness.

Psychological distress in both critical care survivors and their family is commonly recognised following critical illness, comprising anxiety, depression, post‐traumatic stress disorders or complicated grief (Kiekkas et al. 2010). Patient support groups or engaging with psychological interventions have been shown to be beneficial in recovery from critical illness (Booth 2006). In this synthesis, we describe the interconnection of patients and families' physical and psychosocial health, exploring the contribution of physical impairments to poor physical and psychological health.

The lack of coordinated health care provision to facilitate recovery after discharge was experienced by critical care survivors, their families and healthcare professionals. The absence of consensus on how to deliver ICU follow‐up care described by healthcare professionals in this review emphasises the complexity and respective challenges of caring for this population (Connolly et al. 2021). The establishment and funding of ICU follow‐up provision varies globally, and some services remain in their infancy despite clinical guidelines (Cotton 2013).

It is important to understand the specific health problems underlying the physical dependency described within this meta‐ethnography to improve recovery from critical illness. This knowledge will inform healthcare staff on how to support critical care survivors in their recovery. Although we were unable to specify physical impairments in our search terms, the identified studies clearly reported the impact of these impairments on all three groups. One aspect of physical function identified in the included studies was poor musculoskeletal health. This is highly prevalent in critical care survivors, experienced by 59% of patients 6 months after ICU admission (Gustafson et al. 2023). Whilst general experiences of musculoskeletal ill health such as pain and fatigue have emerged within this meta‐ethnography, a specific exploration of these experiences has not been undertaken, so it could be considered as a direction for future research.

There are several strengths to this meta‐ethnography. We took several steps to ensure trustworthiness and credibility in this review: search result screening, study data extraction and quality assessment were undertaken by two independent reviewers; qualitative data extraction was piloted; coding and theme development was discussed regularly within the team; verbatim quotes from papers were used to illustrate points; and GRADE CerQual was used to assess confidence in our findings. The experiences of patients, family and a range of healthcare professionals provide a comprehensive insight into living or providing care support following critical illness. The synthesis also included a global perspective with studies across 13 countries, including an array of healthcare systems.

### 4.1 Limitations

There are some limitations of this meta‐ethnography. The authors had initially planned to focus specifically on physical impairments but during analysis it became clear we were unable to separate these from a holistic biopsychosocial interpretation. The PROSPERO registration has been updated to reflect this change but have highlighted the physical implications of recovery from critical illness within our findings. One study risked significant limitations in recall as a non‐longitudinal study which interviewed patients who had been in ICU up to 11 years of age (Prinjha et al. 2009). Seven studies (Van Beusekom et al. 2016; Davidson and Harvey 2016; Noblit and Hare 1988; France et al. 2019; Page et al. 2021; Booth 2006; Nelderup et al. 2018) included dyadic interviews which may have empowered respondents, but also risk patients and their families hiding their emotions or guilt in order to protect their relative. In the absence of family members to advocate for them, there is a potential that the voices of some critical care survivors are lost. Only one study was from a low to middle income country (King et al. 2022), and none of the studies reported ethnicity, socioeconomic status or other factors such as health literacy that might impact on healthcare engagement.

## 5 Conclusion

This meta‐ethnography is the first to provide an overarching interpretation of the experiences of patients, family members and healthcare professionals of recovering from critical illness. Physical impairments are diverse, commonly preventing patients from thriving and result in patients perceiving themselves as a burden on their families. To overcome the weight of this burden, families try to adapt to their new roles and responsibilities. Our findings highlight an unmet need for follow‐up rehabilitation following critical illness. The impact of specific healthcare problems on physical impairment is poorly understood and should be considered as a discrete focus for future research.

## Author Contributions

E.K., O.G., S.V., M.W., A.W.: Made substantial contributions to conception and design, or acquisition of data, or analysis and interpretation of data. E.K., O.G., S.V., M.W., A.W., F.T.: Involved in drafting the manuscript or revising it critically for important intellectual content. E.K., O.G., S.V., M.W., A.W., F.T.: Given final approval of the version to be published. Each author should have participated sufficiently in the work to take public responsibility for appropriate portions of the content. E.K., O.G., S.V., M.W., A.W., F.T.: Agreed to be accountable for all aspects of the work in ensuring that questions related to the accuracy or integrity of any part of the work are appropriately investigated and resolved.

## Conflicts of Interest

The authors declare no conflicts of interest.

## Supporting information


**Data S1:** jan70189‐sup‐0001‐DataS1.zip.

## Data Availability

Data available on request from the authors. The data that support the findings of this study are available from the corresponding author upon reasonable request.
