# Supplementary material for: Recovery After Critical Illness: A Meta‐Ethnography of Patient, Family and Staff Perspectives
Source: J Adv Nurs. 2025 Oct 2;82(5):4876–94. doi: 10.1111/jan.70189 (PMC13069234; doi:10.1111/jan.70189)
Supplement: Supplementary file 1 — Data S1: jan70189‐sup‐0001‐DataS1.zip. [file JAN-82-4876-s001.zip › Supplementary material_GRADE_final.docx]

**Supplementary material**

**GRADE CERQual: Summary of review findings.**

| Summary of review finding | Studies contributing to the review finding | CERQual assessment of confidence in the evidence | Explanation of CERQual assessment |
| --- | --- | --- | --- |
| Finding 1: Despite being grateful to have survived their critical illness, patients struggled with the uncertainty of their ongoing recovery after hospital discharge. In particular, the loss of independence with their mobility and function was impactful. This led to a sense of vulnerabilities such as feeling bitter, bored and feeling a lack of purpose. | (1–26) | High confidence | No concerns regarding relevance and coherence. Very minor concerns regarding methodological limitations and adequacy. |
| Finding 2: Patients and family struggled to access support for rehabilitation. They felt uncared for, and it was a stressful time period. Staff reported the lack of operational consistency in the provisional of follow-up care, in particular with information. | (1–30) | Moderate | Moderate concerns regarding methodological limitations and relevance. No/minor concerns regarding coherence and adequacy. |
| Finding 3: Patients felt they were a burden, and family respectively felt the toll of this. However, family members were steadfast in their commitment to care. | (1–4,6–9,11–23,28,30–34) | Moderate | Moderate concerns regarding methodological limitations and adequacy. No/minor concerns regarding relevance and coherence. |
| Finding 4: Patients described the physical impairments they endured, and that persisted. They had lost confidence and trust in their bodies. | (1–17,17–27) | Moderate | Moderate concerns regarding methodological limitations and adequacy. No/minor concerns regarding relevance and coherence. |

1. Abdalrahim M, Zeilani R. Jordanian survivors’ experiences of recovery from critical illness: A qualitative study. Int Nurs Rev. 2014 Dec 1;61.

2. Ågård AS, Egerod I, Tønnesen E, Lomborg K. Struggling for independence: A grounded theory study on convalescence of ICU survivors 12 months post ICU discharge. Intensive Crit Care Nurs. 2012 Apr;28(2):105–13.

3. Ågård AS, Egerod I, Tønnesen E, Lomborg K. From spouse to caregiver and back: a grounded theory study of post‐intensive care unit spousal caregiving. J Adv Nurs. 2015 Aug;71(8):1892–903.

4. Alexandersen I, Haugdahl HS, Stjern B, Paulsby TE, Lund SB, Haugan G. ‘I want to get back!’ A qualitative study of long-term critically ill patients’ inner strength and willpower: Back home after long-term intensive care. J Clin Nurs. 2021;30(19–20):3023–35.

5. Allum L, Connolly B, McKeown E. Meeting the needs of critical care patients after discharge home: a qualitative exploratory study of patient perspectives. Nurs Crit Care. 2018 Nov;23(6):316–23.

6. Calkins K, Kako P, Guttormson J. Patients’ experiences of recovery: Beyond the intensive care unit and into the community. J Adv Nurs. 2021 Apr;77(4):1867–77.

7. Cox CE, Docherty SL, Brandon DH, Whaley C, Attix DK, Clay AS, et al. Surviving Critical Illness: The Acute Respiratory Distress Syndrome as Experienced by Patients and Their Caregivers. Crit Care Med. 2009 Oct;37(10):2702–8.

8. Czerwonka AI, Herridge MS, Chan L, Chu LM, Matte A, Cameron JI. Changing support needs of survivors of complex critical illness and their family caregivers across the care continuum: a qualitative pilot study of Towards RECOVER. J Crit Care. 2015;30(2):242–9.

9. Deacon KS. Re-building life after ICU: A qualitative study of the patients’ perspective. Intensive Crit Care Nurs. 2012 Apr;28(2):114–22.

10. Eaton TL, Lewis A, Donovan HS, Davis BC, Butcher BW, Alexander SA, et al. Examining the needs of survivors of critical illness through the lens of palliative care: A qualitative study of survivor experiences. Intensive Crit Care Nurs. 2023 Apr 1;75:103362.

11. Hanifa ALB, Glæemose AO, Laursen BS. Picking up the pieces: Qualitative evaluation of follow-up consultations post intensive care admission. Intensive Crit Care Nurs. 2018 Oct;48:85–91.

12. Jensen JF, Overgaard D, Bestle MH, Christensen DF, Egerod I. Towards a new orientation: a qualitative longitudinal study of an intensive care recovery programme. J Clin Nurs. 2017 Jan;26(1–2):77–90.

13. Kang J, Jeong YJ. Embracing the new vulnerable self: A grounded theory approach on critical care survivors’ post-intensive care syndrome. Intensive Crit Care Nurs. 2018 Dec 1;49:44–50.

14. Karlsson V, Bergbom I, Ringdal M, Jonsson A. After discharge home: a qualitative analysis of older ICU patients’ experiences and care needs. Scand J Caring Sci. 2016 Dec;30(4):749–56.

15. Nelderup M, Simonsson A, Samuelson K. Intensive care survivors’ experiences of recovery after hospital discharge: A qualitative interview study. Nord J Nurs Res. 2018 Dec 1;38(4):196–203.

16. Page P, Simpson A, Reynolds L. Constructing a grounded theory of critical illness survivorship: The dualistic worlds of survivors and family members. J Clin Nurs. 2019;28(3–4):603–14.

17. Palesjö C, Nordgren L, Asp M. Being in a critical illness‐recovery process: a phenomenological hermeneutical study. J Clin Nurs. 2015 Dec;24(23–24):3494–502.

18. Pattison N, Dolan S. Exploring patients’ experiences of a nurse-led follow-up service after critical care. Nurs Times. 2009 May 1;105(19):16–9.

19. Pattison N, O’Gara G, Rattray J. After critical care: Patient support after critical care. A mixed method longitudinal study using email interviews and questionnaires. Intensive Crit Care Nurs. 2015 Aug 1;31(4):213–22.

20. Prinjha S, Field K, Rowan K. What patients think about ICU follow-up services: a qualitative study. Crit Care. 2009 Apr 1;13(2):R46.

21. Thurston LM, Milnes SL, Hodgson CL, Berkovic DE, Ayton DR, Iwashyna TJ, et al. Defining patient-centered recovery after critical illness – A qualitative study. J Crit Care. 2020 Jun 1;57:84–90.

22. Vester LB, Holm A, Dreyer P. Patients’ and relatives’ experiences of post‐ICU everyday life: A qualitative study. Nurs Crit Care. 2022 May;27(3):392–400.

23. Walker W, Wright J, Danjoux G, Howell SJ, Martin D, Bonner S. Project Post Intensive Care eXercise (PIX): A qualitative exploration of intensive care unit survivors’ perceptions of quality of life post-discharge and experience of exercise rehabilitation. J Intensive Care Soc. 2015 Feb 1;16(1):37–44.

24. Juuso P, Engström Å, Strömbäck U, Andersson M, Nordin A. Getting Back on Track: Meanings of Recovery After Critical Illness Caused by COVID-19. SAGE Open Nurs. 2024 Jan;10:23779608241282922.

25. O’Neill B, Green N, Blackwood B, McAuley D, Moran F, MacCormac N, et al. Recovery following discharge from intensive care: What do patients think is helpful and what services are missing? Amaravadi SK, editor. PLOS ONE. 2024 Mar 18;19(3):e0297012.

26. Paton M, Le Maitre C, Berkovic D, Lane R, Hodgson CL. The impact of critical illness on patients’ physical function and recovery: An explanatory mixed-methods analysis. Intensive Crit Care Nurs. 2024 Apr;81:103583.

27. Geense WW, de Graaf M, Vermeulen H, van der Hoeven J, Zegers M, van den Boogaard M. Reduced quality of life in ICU survivors - the story behind the numbers: A mixed methods study. J Crit Care. 2021 Oct 1;65:36–41.

28. Danielis M, Garau A, Molaro D, Gentilini S, Rosset M, Giorgino S, et al. Navigating post-ICU care: understanding family members’ experiences - a qualitative study. Health Psychol Behav Med. 2024 Dec 31;12(1):2415394.

29. Zhang F, Chen Z, Xue D, Zhang R, Cheng Y. Barriers and facilitators to offering post‐intensive care follow‐up services from the perspective of critical care professionals: A qualitative study. Nurs Crit Care. 2024 Jul;29(4):682–94.

30. Wendlandt B, Edwards T, Hughes S, Gaynes BN, Carson SS, Hanson LC, et al. Novel Definitions of Wellness and Distress among Family Caregivers of Patients with Acute Cardiorespiratory Failure: A Qualitative Study. Ann Am Thorac Soc. 2024 May;21(5):782–93.

31. Frivold G, Slettebø Å, Dale B. Family members’ lived experiences of everyday life after intensive care treatment of a loved one: a phenomenological hermeneutical study. J Clin Nurs. 2016 Feb;25(3–4):392–402.

32. Johansson I, Fridlund B, Hildingh C. Coping strategies of relatives when an adult next-of-kin is recovering at home following critical illness. Intensive Crit Care Nurs. 2004 Oct 1;20(5):281–91.

33. Nelderup M, Samuelson K. Experiences of partners of intensive care survivors and their need for support after intensive care. Nurs Crit Care. 2020 Jul;25(4):245–52.

34. Sevin CM, Boehm LM, Hibbert E, Bastin AJ, Jackson JC, Meyer J, et al. Optimizing Critical Illness Recovery: Perspectives and Solutions From the Caregivers of ICU Survivors. Crit Care Explor. 2021 May 12;3(5):e0420.
