# Supplementary material for: Recovery After Critical Illness: A Meta‐Ethnography of Patient, Family and Staff Perspectives
Source: J Adv Nurs. 2025 Oct 2;82(5):4876–94. doi: 10.1111/jan.70189 (PMC13069234; doi:10.1111/jan.70189)
Supplement: Supplementary file 1 — Data S1: jan70189‐sup‐0001‐DataS1.zip. [file JAN-82-4876-s001.zip › Supplementary_emerge_final.docx]

| No. | Criteria Headings | Reporting Criteria | Pages |
| --- | --- | --- | --- |
| Phase 1 – Selecting meta-ethnography and getting started | | | |
| 1 | Rationale and context for the meta-ethnography | Describe the gap in research or knowledge to be filled by meta-ethnography, and the wider context of the meta-ethnography | 2-3 |
| 2 | Aim(s) of the meta-ethnography | Describe the meta-ethnography aim(s) | 2 |
| 3 | Focus of the meta-ethnography | Describe the meta-ethnography review question(s) (or objectives) | 3 |
| 4 | Rationale for using meta-ethnography | Explain why meta-ethnography was considered the most appropriate qualitative synthesis methodology | - |
| Phase 2 – Deciding what is relevant | | | |
| 5 | Search strategy | Describe the rationale for the literature search strategy | 3 |
| 6 | Search processes | Describe how the literature searching was carried out and by whom | 3 |
| 7 | Selecting primary studies | Describe the process of study screening and selection, and who was involved | 4 |
| Findings |  |  |  |
| 8 | Outcome of study selection | Describe the results of study searches and screening | 4 |
| Phase 3 – Reading included studies | | | |
| 9 | Reading and data extraction approach | Describe the reading and data extraction method and processes | 4 |
| Findings |  |  |  |
| 10 | Presenting characteristics of included studies | Describe characteristics of the included studies | 5 |
| Phase 4 – Determining how studies are related | | | |
| Methods | | | |
| 11 | Process for determining how studies are related | Describe how the methods and processes for determing how the included studies are related: - Which aspects of studies are compared AND – How the studies were compared | 5 |
| Findings | | | |
| 12 | Outcome of relating studies | Describe how studies relate to each other | 5 |
| Phase 5 – Translating studies into one another | | | |
| Methods | | | |
| 13 | Process of translating studies | Describe the methods to translation:   - Describe steps taken to preserve the context and meaning of the relationships between concepts within and across studies – Describe how the reciprocal and refutational translations were conducted – Describe how potential alternative interpretations or explanations were considered in the translations | 5 |
| Findings | | | |
| 14 | Outcome of translation | Describe the interpretative findings of the translation | 5-10 |
|  |  |  |  |
| Phase 6 – Synthesizing translations | | | |
| Methods | | | |
| 15 | Synthesis process | Describe the methods used to develop overarching concepts (“synthesised translations”) Describe how the alternative interpretations or explanations were considered in the synthesis | 5 |
| Findings | | | |
| 16 | Outcome of synthesis process | Describe the new theory, conceptual framework, model, configuration, or interpretation of data developed from the synthesis | 10 |
| Phase 7 – Expressing the synthesis | | | |
| Discussion | | | |
| 17 | Summary of findings | Summarize the main interpretation findings of the translation and synthesis and compare to existing literate | 11-12 |
| 18 | Strengths, limitations and reflexivity | Reflect on and describe the strengths and limitations of the synthesis:   - Methodological aspects – for example, the describe how the synthesis findings were influenced by the nature of the included studies and how the meta-ethnography was conducted. Reflexivity – for example, the impact of the research team on the synthesis findings | 3, 12 |
| 19 | Recommendations and conclusions | Describe the implications of the synthesis | 12 |
